# Biomechanical investigation of external fixators for the treatment of supracondylar humerus fractures

**DOI:** 10.1186/s13018-020-01815-2

**Published:** 2020-07-28

**Authors:** Christoph Castellani, Holger Till, Annelie-Martina Weinberg

**Affiliations:** 1 Department of Pediatric and Adolescent Surgery, Medical University of Graz, Graz, Austria; 2 Department of Orthopaedics and Traumatology, Medical University of Graz, Graz, Austria

In children, supracondylar humerus lesions rank among the most common fractures. Closed reduction and percutaneous pinning remains the gold standard of treatment (summarized in [1]). Elastic stabile intramedullary nailing (ESIN) has been proposed as alternative. However, these techniques may not provide adequate stability especially in case of comminution zones or oblique fractures. Thus, external fixation (EF) with various technical configurations has been suggested as alternative. Additionally, EF may have advantages in open fractures with contamination or impending compartment syndrome. In the past, EF has been the subject of biomechanical stability testing. In this regard, Li et al. published an analysis comparing different k-wire configurations and external fixation in your Journal in 2018 [2]. They concluded: “External fixator could provide enough stability for pediatric supracondylar humerus fractures without the injury of the ulnar nerve. Besides, it could enhance the rotational stiffness of the construct in rotation loading to avoid the complication of cubitus varus” [2]. In the discussion section, they state that “there have been no published reports of biomechanical analysis in the external fixator in supracondylar humerus fractures” [2].

This statement of Li et al. being the first to report biomechanical data on EF is not correct. Already in 2007, Weinberg et al. have published a study with a biomechanical comparison of crossed pinning, elastic stabile intramedullary nailing (ESIN), and two different configurations of EF in a cadaver model in PubMed listed Clinical Biomechanics [3].

In our study, we found no significant differences for the 4 methods tested in quasi-static flexional, extensional, and torsional loading [3]. In contrast to Li et al., specimen in our tests was also subjected to cyclic loading simulating movement during fracture healing. In this test, EF had worse outcomes with higher irreversible axial deformations compared to crossed k-wires and ESIN. The irreversible angular deformation was highest for the EF using k-wires followed by ESIN and the EF with Schanz screws and crossed pinning [3].

Regarding other publications, a study by Kamara et al. compared k-wire osteosynthesis with different pin configurations, ESIN and EF with Schanz screws in different static loading conditions [4]. They described that ESIN provided the best overall stability in proximal fractures while pinning was superior to stabilize fractures in the distal supracondylar region. Regarding pin placement, they found two lateral and one medial pin to be the most stable pin configuration. Their data reflects our findings with good stability of crossed pinning in distal fractures. Finally, Hohloch et al. reported the superior stability of ulnar over radial anti-rotation wires in EF with Schanz screws under static loading conditions [1].

Possible reasons for the discrepancy between the different biomechanical studies may be attributed to the experimental model: cadaver or synthetic bone, fracture type, and mode of biomechanical testing. Especially, the fracture type will have impact on the test results of EF. While we tested our specimens with a fracture gap simulating fracture comminution, other authors used models without gaps. This could explain the different data regarding EF: if a compression of the fracture gap is applied it is superior to other methods, if a gap remains it is less stable than crossed pinning or ESIN.

We congratulate Li and co-workers to their work, but would recommend being more careful with their statement being the first to report biomechanical data on EF in supracondylar fractures because extensive experimental work has already been published regarding this issue a decade before.

## Data Availability

Not applicable.
